# Acute Serious Thrombocytopenia Associated with Intracoronary Tirofiban Use for Primary Angioplasty

**DOI:** 10.1155/2014/190149

**Published:** 2014-03-10

**Authors:** Mustafa Yurtdaş, Yalin Tolga Yaylali, Nesim Aladağ, Mahmut Özdemir, Memiş Hilmi Atay

**Affiliations:** ^1^Department of Cardiology, Van Region Training and Research Hospital, 65100 Van, Turkey; ^2^Department of Cardiology, Pamukkale University, 20070 Denizli, Turkey; ^3^Department of Hematology, Van Region Training and Research Hospital, 65100 Van, Turkey

## Abstract

Tirofiban, a specific glycoprotein IIb/IIIa inhibitor, may cause extensive thrombocytopenia with an incidence of 0.2% to 0.5%. We report the case of a 50-year-old man who developed thrombocytopenia after tirofiban use (both intracoronary and peripheral) over hours and the successful management of this complication after primary percutaneous coronary intervention for acute ST-segment elevation myocardial infarction.

## 1. Introduction 

Glycoprotein IIb/IIIa inhibitors (GPIs) are commonly employed in treating patients who have unstable angina, non-ST, and ST-segment elevation myocardial infarction (STEMI), as well as in combination with angioplasty with or without stent placement [1]. Tirofiban, a specific and nonpeptide GPI, competitively inhibits the platelet fibrinogen receptor and may lead to severe thrombocytopenia with an incidence of 0.2% to 0.5% [2]. In this report, we describe a case of acute serious thrombocytopenia after 4 h of tirofiban administration in a patient in whom primary percutaneous coronary intervention (PCI) was performed for acute anterior STEMI.

## 2. Case Report

A 50-year-old man presented with an acute anterior STEMI. Initial laboratory tests showed a normal complete blood count (CBC) including platelet count (265 × 10^9^/L) at the emergency department. He reports no history of bleeding disorders, hematologic and renal problems, or heparin exposure. He immediately underwent PCI after pretreatment with 300 mg of aspirin and 600 mg of clopidogrel and 10.000 IU of intravenous unfractionated heparin. Coronary angiography showed the totally occluded left anterior descending artery (LAD). After predilatation, a large and fresh thrombus was seen. We first administered tirofiban via intracoronary route at a dose of 10 µg/kg followed by peripheral intravenous infusion at 0.15 µg/kg/min and then implanted a coronary 4.5 × 18 mm bare metal stent into LAD. A combination therapy of aspirin, clopidogrel, enoxaparin, and tirofiban infusion was given to the patient. Approximately 4 h after the PCI, areas of petechiae and ecchymoses were observed around the sternum and on both legs. The patient's platelet count was detected to be 5 × 10^9^/L (Table 1). Checkup on the peripheral smear of a blood sample validated the extensive lack of platelets with no clustering. All antiplatelet drugs including tirofiban were immediately discontinued, and the patient was treated with Ig G infusion in order to achieve a quick recovery. There was a very slight rise on day 1, with improvement beginning after day 2 and counts surpassing 100 × 10^9^/L on day 4 (Table 1). During this time, the patient was supported with Ig G infusion. Clopidogrel and aspirin were restarted when the platelet count surpassed 50 × 10^9^/L. In the meantime, the patient did demonstrate neither any evidence of bleeding related adverse events nor hemodynamic instability.

## 3. Discussion 

Thrombocytopenia is defined as a platelet count below the normal range for the population [3]. An accumulating evidence suggests that there is a clear association between GPI use and thrombocytopenia [4–8]. Five pictures of thrombocytopenia caused by GPIs have been described: (i) acute severe thrombocytopenia within 12 h of first exposure (platelets <10 × 10^9^/L), (ii) acute thrombocytopenia within 12 h of second exposure, (iii) delayed thrombocytopenia (five to seven days after treatment), (iv) anaphylaxis after first or second exposure, and (v) pseudothrombocytopenia [7]. The main mechanism responsible for GPI-induced thrombocytopenia has been proposed to be drug-dependent antibodies that are naturally taking place or induced by previous exposure to the drug [2, 7, 8]. The differential diagnosis for some other drug-induced thrombocytopenias should be punctiliously made. Heparin-induced thrombocytopenia (HIT) type I tends to occur within minutes to hours of postexposure in those who have received heparin therapy within the past 6 months and is usually mild and asymptomatic [9]. In HIT type II, the mechanism is immunologic in origin, and thrombocytopenia typically occurs approximately 5 days after initiation of treatment in patients without prior exposure to heparin [9]. Although we did not search for heparin-dependent antibodies, we consider that the acute severe thrombocytopenia observed in our case was very unlikely to be heparin induced, because our patient had no prior exposure to heparin. Aspirin and clopidogrel very seldom give rise to isolated acute severe thrombocytopenia [10]. This patient had no prior history of the use of these drugs and received aspirin and clopidogrel immediately before primary PCI and these drugs were resumed 2 days after thrombocytopenia was resolved without further drop in platelet counts. Clopidogrel may cause thrombotic thrombocytopenic purpura (TTP), which is characterized by microangiopathic hemolytic anemia, thrombocytopenia, neurologic abnormalities, fever, and renal dysfunction [10, 11]. In our case, we observed the thrombocytopenia only, not the other clinical findings of TTP. In addition, clopidogrel-associated TTP usually develops in the first 2 weeks after initiation of treatment. Tirofiban was given 4 h prior to the development of thrombocytopenia. The recovery was completed and sustained after tirofiban was discontinued. Tirofiban was the only drug which was discontinued and never resumed, whereas aspirin and clopidogrel were resumed without any problems. The patient sustained a normal platelet count. Thus, we excluded aspirin and clopidogrel as a cause for isolated thrombocytopenia. The patient never had re-exposure to tirofiban later. Therefore, our case had most probably tirofiban-induced thrombocytopenia. When thrombocytopenia is diagnosed, it is necessary to interrupt tirofiban therapy. With termination of tirofiban infusion, platelet counts usually return to normal levels over a period of 2 to 5 days (as in our case). During this time, treatment with steroids, Ig infusion, and platelet transfusion may be life saving [2–7]. In pseudothrombocytopenia, there is neither in vivo thrombocytopenia nor increased aggregation. This artifactual clumping of platelets should be ruled out by manually testing peripheral blood smears or repeating the platelet count in blood samples anticoagulated with citrate [2, 3]. To our knowledge, there is no report in which tirofiban was found to be related to pseudothrombocytopenia.

In conclusion, our report illustrates the importance of awareness of the life threatening thrombocytopenia associated with intracoronary tirofiban use. The measurement of platelet count before and early after the initiation of tirofiban treatment is very important because the tirofiban-induced thrombocytopenia can be resolved by the interruption of tirofiban infusion and early supportive treatment.

## Figures and Tables

**Table 1:** Platelet counts at baseline and after the percutaneous coronary intervention.

Sample time	Platelet count (×10^9^/L)
Baseline	265
4 h	5
12 h	5.7
24 h	11
32 h	14
48 h	25
64 h	34
72 h	62
86 h	110
96 h	153
